# ProtDataTherm: A database for thermostability analysis and engineering of proteins

**DOI:** 10.1371/journal.pone.0191222

**Published:** 2018-01-29

**Authors:** Hassan Pezeshgi Modarres, Mohammad R. Mofrad, Amir Sanati-Nezhad

**Affiliations:** 1 BioMEMS and Bioinspired Microfluidic Laboratory, Department of Mechanical and Manufacturing Engineering, University of Calgary, Calgary, Alberta, Canada; 2 Molecular Cell Biomechanics Laboratory, Departments of Bioengineering and Mechanical Engineering, University of California Berkeley, 208A Stanley Hall, Berkeley, CA, United States of America; 3 School of Biological Sciences, Institute for Research in Fundamental Sciences (IPM), Tehran, Iran; 4 Physical Biosciences Division, Lawrence Berkeley National Lab, Berkeley, CA, United States of America; 5 Center for BioEngineering Research and Education, University of Calgary, Alberta, Canada; Russian Academy of Medical Sciences, RUSSIAN FEDERATION

## Abstract

Protein thermostability engineering is a powerful tool to improve resistance of proteins against high temperatures and thereafter broaden their applications. For efficient protein thermostability engineering, different thermostability-classified data sources including sequences and 3D structures are needed for different protein families. However, no data source is available providing such data easily. It is the first release of ProtDataTherm database for analysis and engineering of protein thermostability which contains more than 14 million protein sequences categorized based on their thermal stability and protein family. This database contains data needed for better understanding protein thermostability and stability engineering. Providing categorized protein sequences and structures as psychrophilic, mesophilic and thermophilic makes this database useful for the development of new tools in protein stability prediction. This database is available at http://profiles.bs.ipm.ir/softwares/protdatatherm. As a proof of concept, the thermostability that improves mutations were suggested for one sample protein belonging to one of protein families with more than 20 mesophilic and thermophilic sequences and with known experimentally measured ΔT of mutations available within ProTherm database.

## Introduction

Thermophilic and hyper thermophilic microorganisms have become attractive to scientists specifically after reporting the microorganisms living at temperatures higher than 75°C (1). The extracted enzymes from such high temperature tolerating microorganisms have been studied to understand modulating factors of their improved thermostability and then to use it as a guidance for improving thermostability of proteins with lower thermal stability for biotechnological applications [1]. The knowledge about the preferred living temperature of microorganisms can help to approximate thermostability criteria of their expressed proteins and a direct relationship between the growth temperature of microorganisms and the melting point of their corresponding proteins [2]. Currently available data on homologous proteins are valuable for engineering of proteins to gain higher stability by for example introducing more salt-bridges or strengthening the hydrophobic cores within protein structure [3]. Although structure-based protein engineering, known as rational engineering or rational design, is the most popular methodology for thermostability engineering of proteins, the limited number of available protein structures is still a challenge to prevalent utilization of the methodology [4]. On the other hand, because of modern advances in DNA sequencing technologies, the number of sequenced proteins belonging to different families is growing rapidly [3, 5]. Advances in applications of protein sequences for protein engineering could assist the existing routine structure-based rational methods. The consensus concept (CC) is the most popular sequence-based protein engineering approach to extract thermo-stabilizing mutations out of homologous sequences [6–17]. In CC approach, a multiple sequence alignment (MSA) is first made and then non-consensus residues are substituted by the most frequently occurring amino acids [5]. However, there is no guarantee that all suggested mutations induced by CC approach can increase thermostability [9, 14, 16, 18]. To detect thermo-stabilizing mutations with higher probability, one can take the advantage of comparing the target sequence with homologues tolerant at higher temperatures [3]. To make it feasible for different families of proteins, one needs to have access to other proteins from the same family with a higher thermal stability. However, the main challenge using this method is the difficulty in finding homologues with a label showing the thermostability category. To overcome this challenge, we developed a comprehensive database that contains protein sequences that belongs to different microorganisms and clustered based on the Pfam ID. The user can find the Pfam ID of a protein of interest and find its homologues, categorized as psychrophilic, mesophilic and thermophilic. In addition to sequences, PDB IDs are also provided if a 3D structure is available for the Pfam ID of interest.

## Materials and methods

**Fig 1:**
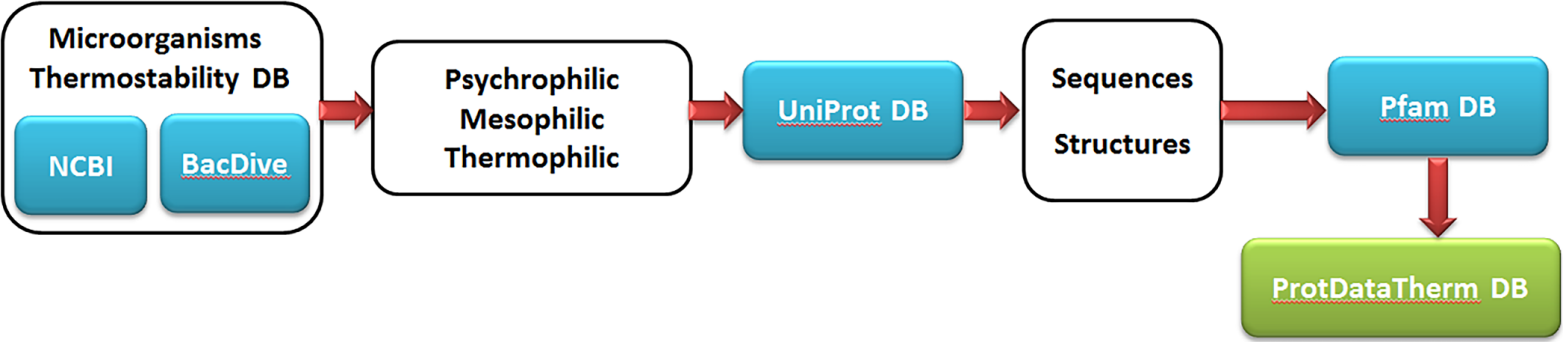
Flowchart of the database formation.

First, a database was made for microorganisms such that each microorganism is categorized based on its growth temperature (GT) using BacDive [19] and NCBI [20] databases. For every microorganism, all available sequences with their corresponding sequence information, including Pfam ID [21] and PDB ID [22], if available, were obtained from UniProt database [23]. All the process was conducted using python programming language [24], incorporating Biopython module [25] (**Fig 1**).

In our database, all protein sequences have two labels: Pfam IDs and thermostability category. To facilitate the use of the database for thermostability analysis and engineering, sequences are clustered based on their Pfam IDs. For each Pfam ID cluster, we can find proteins from the same family labeled with their thermostability category. Therefore, for a target protein sequence, the user can find the corresponding Pfam ID from the Pfam database [21] and uses the Pfam ID as the primary input to search over the database. For each Pfam ID family, we categorized sequences based on their Uniprot IDs as psychrophilics (GT< 20°C), mesophilics (20°C < GT < 40°C), and thermophilic (40°C <GT). For each protein family, the available PDB structures are shown and categorized like sequences. All sequence IDs, protein family IDs, and PDB IDs, are UniProt, Pfam and RCSB IDs, respectively.

For the case study, first, Pfams containing more than 20 mesophilic and thermophilic sequences were found. Then, for pattern analysis, the AXB patterns were considered in each sequence where A and B can be any of 20 standard amino acids and X is a separation number between 0 and 10. Therefore, A0B means all double amino acid compositions that are subsequent like VE, and A1B patterns are all double amino acid compositions that there is one amino acid between them. For example, all patterns with Ala as the first amino acid, Val as the second, and with only one amino acid spacing between Ala and Val from the 20 standard amino acids are considered as A1V. The condition 0 = <X = <10 was used for the spacing values. Furthermore, for any of sequences in mesophilic and thermophilic sequences, the number of occurring AXB patterns were counted and saved for each sequence. Finally, we have a group of data for both mesophilic and thermophilic sequences with the corresponding patterns. Therefore, for a given AXB (e.g. V4H pattern), there is one group of numbers for mesophilic and thermophilic categories with their corresponding average number. The Rank Sum test with critical p-value of 0.05 was used to detect AXB patters and distinguish mesophilic sequences from thermophilic sequences.

## Results and discussion

**Fig 2:**
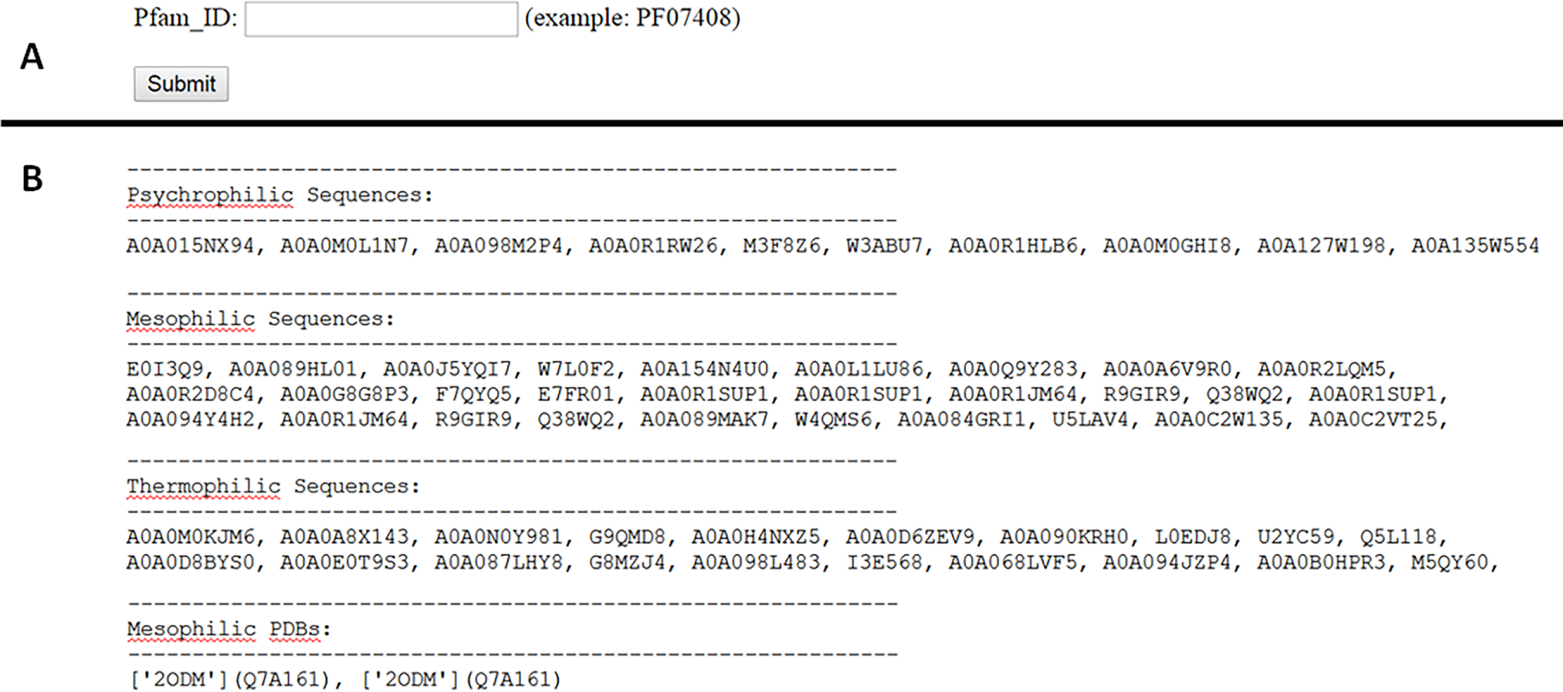
The view of the webpage. A) Users can enter the Pfam ID as input at the first page. B), All available sequences and structures are presented for different classes at the result page.

A PHP webpage is designed as the user interface to access the database. The user can find the Pfam ID for a protein of interest (e.g. using Pfam database) and search it in the first page of the website (**Fig 2**, panel A). The results are then presented in the next page including all available sequences and structures within the database for the submitted Pfam ID (**Fig 2**, panel B). The database contains more than 14 million protein sequences and PDB structures for 9962 protein family, categorized based on their thermal stability as psychrophilic, mesophilic and thermophilic (**Table 1**). Totally, there are 14155392 protein sequences and 30950 PDB structures available in the database. For 957 members of protein families there is at least one PDB structure available for a thermophilic protein that can be used for structural comparison between mesophilic and thermophilic proteins (**Table 1**). In addition, for 3355 protein families there are at least 20 sequences belonging to thermophilic proteins as well as 3046 protein families with at least 20 sequences belonging to psychrophilic proteins. For such protein families, we can use amino acid content comparison between psychrophilic/mesophilic and mesophilic/thermophilic proteins to gain protein family-based specific knowledge of thermostability modulating factors.

**Table 1:** The distribution of protein sequences and structures over the three classes of thermostability.

Mesophilic sequences	13111756
Thermophilic sequences	661072
Psychrophilic sequences	382564
Mesophilic structures	23069
Thermophilic structures	7741
Psychrophilic structures	140
Pfams with at least one Mesophilic structure	2306
Pfams with at least one Thermophilic structure	957
Pfams with at least one Psychrophilic structure	82
Pfams with at least 20 Thermophilic sequence	3355
Pfams with at least 20 Psychrophilic sequence	3046

### Other databases

Two databases, namely PGTdb [26] and Protherm [27], are presently available to provide data concerning protein thermostability. To the knowledge of authors, the PGTdb database is not presently available while it was the only resource that could provide experimental information about thermostability classification of protein sequences based on GT of their corresponding organisms (psychrophilic, mesophilic and thermophilic). On the other hand, ProTherm database provides thermodynamics data for mutagenesis but only for a limited number of proteins. Our database contains much higher number of microorganisms, protein sequences and PDB structures. This database categorizes all the sequences for different Pfam families according to their thermostability criteria and provides easier access to the needed data for analysis and engineering of protein families.

### Case study: Pattern recognition for protein engineering

One important goal of all thermostability analysis is to understand how one can take advantage of the knowledge from analysis of the differences between two categories, engineer mesophilics by minimum number of mutations, and enhance protein thermostability towards thermophilic sequences. Here, as a case study, we selected a protein belonging to one of those protein families with more than 20 mesophilic and thermophilic sequences where its ΔT of mutations is experimentally available within ProTherm database. In the ProTherm database, ribonuclease H from Escherichia Coli (strain K12) (with PDB_ID of 2RN2, solved using X-ray diffraction, resolution 1.48Å) was selected. Ribonuclease belongs to Pfam ID of PF00075, with the reported ΔT upon mutation using thermal experiments and is amongst the proteins with the highest number of reported thermodynamic measurements for the effect of mutations on its stability.

An algorithm **(Algorithm 1)** is designed to suggest thermostability improving mutations: for all AXB patterns with meaningful population difference between mesophilic and thermophilic sequences in the family (Pfam ID of PF00075) (see methods for definition of meaningful population difference), we chose those AXB patterns that have a higher average number of repeats than mesophilic within thermophilic category. We then found AXY patterns in the target sequence (ribonuclease H from Escherichia Coli) that the Y is not equal to B in the pattern. For these selected patterns, we suggest Y→B mutation. The same approach was used for ZXB to suggest Z→A mutations. If the mutation was available in the ProTherm database, the ΔT value was checked. If ΔT > 0, the suggested mutation was considered as a successful thermostability improving suggestion and if ΔT < 0, it was defined as a failed suggestion. The results are shown in **Table 2** where 72% of the suggested mutations can improve thermostability. This result confirms that the proposed method can be considered as a sequence-based thermostability engineering method only if we have categorized sequences as thermophilic and mesophilic for protein family of the target proteins. The accuracy of the suggested mutations for thermostability engineering is expected to be improved over such a database by recruiting more complicated methods like machine learning techniques. However, further studies with incorporation of more proteins from diverse range of protein families should be conducted to better evaluate the accuracy of this method.

**Algorithm 1. Thermostability improving mutation suggestion algorithm**.

**Input.** Protein sequence, P-fam ID, and thermophilic and mesophilic

distinguishing AXB patterns for the P-fam ID

**Output.** Mutation list

**for** all *AXB* patterns for the P-fam ID **do:**

    **if** ${AXB}_{average}^{thermophilic}>{AXB}_{average}^{mesophilic}$ **then:**

      find *AXY* or *ZXB* patterns in the target sequence where Y is

      not B or Z is not A

      add Y → B or Z → A to mutation list

    **end**

**end**

**return** mutation list

**Table 2:** Ave_The: Average of the number of patterns for thermophilic sequences, Ave_Mes: Average of the number of patterns for mesophilic sequences.

Pattern	Positions on Sequence	Mutation	ΔT	P_value	Ave_The	Ave_Mes
ER	61 E, H 62	H 62 R	1.3	0.0032	1.782	1.474
LR	74 V, R 75	V 74 L	3.7	0.0106	1.773	1.492
LE	134 D, E 135	D 134 L	5.5	0.0007	1.918	1.437
NK	95 K, K 96	K 95 N	3.2	0.00841	1.951	1.43
SI	52 A, I 53	A 52 S	-5.8	0.04146	1.579	1.21
SG	10 D, G 11	D 10 S	9.2	0.0083	1.836	1.63
KI	52 A, I 53	A 52 K	19.5	0.0398	2.385	1.69
EG	10 D, G 11	D 10 E	3.8	0.012	1.74	1.376
F1E	8 F, D 10	D 10 E	3.8	0.0199	1.463	1.242
F1S	8 F, D 10	D 10 S	9.2	0.0186	1.767	1.284
C2N	41 R, N 44	R 41 C	1.6	0.0002	1.282	1.052
A2Y	70 D, Y 73	D 70 A	3.8	0.004	1.647	1.304
E2Y	70 D, Y 73	D 70 E	1.8	0.0331	1.583	1.12
E2C	10 D, C 13	D 10 E	3.8	0.008	1.409	1
L2N	49 L, A 52	A 52 N	-5.9	0.0323	1.617	1.301
L2N	67 L, D 70	D 70 N	5.5	0.0323	1.617	1.301
V2K	119 E, K 122	E 119 V	2.7	0.0379	1.635	1.264
N3I	130 N, D 134	D 134 I	4.6	0.0281	1.667	1.246
N3N	130 N, D 134	D 134 N	6.4	0.0004	1.658	1.265
N3E	130 N, D 134	D 134 E	3.1	0.0353	1.757	1.557
N3V	130 N, D 134	D 134 V	4.1	0.0031	1.541	1.299
N3V	70 D, V 74	D 70 N	5.5	0.0031	1.541	1.299
R3V	91 K, K 95	K 91 R	0.5	0.0005	1.554	1.26
V3Y	24 A, Y 28	A 24 V	3.2	0.0419	1.638	1.136
E3V	48 E, A 52	A 52 V	7.8	0.0133	2.023	1.852
E3V	64 E, S 68	S 68 V	1.9	0.0133	2.023	1.852
E3V	70 D, V 74	D 70 E	1.8	0.0133	2.023	1.852
E3V	94 D, V 98	D 94 E	-1.2	0.0133	2.023	1.852
Y3L	52 A, L 56	A 52 Y	-7.6	0.0146	1.636	1.082
C4E	52 A, E 57	A 52 C	2.5	0.0175	1.4	1
V4Y	68 S, Y 73	S 68 V	1.9	0.0162	1.528	1.079
N4R	70 D, R 75	D 70 N	5.5	7.34E-09	1.587	1.155
N4K	130 N, E 135	E 135 K	-0.8	6.92E-05	2.329	1.678
N4E	52 A, E 57	A 52 N	-5.9	0.0127	1.664	1.317
Q5N	4 Q, D 10	D 10 N	6.8	0.0361	1.696	1.16
E5N	64 E, D 70	D 70 N	5.5	0.00257	1.615	1.36
E5V	10 D, N 16	D 10 E	3.8	0.0025	1.615	1.3
E5N	94 D, N 100	D 94 E	-1.2	0.0025	1.615	1.36
R5P	46 R, A 52	A 52 P	-5.4	0.0499	1.37	1.217
R5P	91 K, P 97	K 91 R	0.5	0.0499	1.37	1.217
R5I	46 R, A 52	A 52 I	6.2	0.0299	1.429	1.206
R5Y	46 R, A 52	A 52 Y	-7.6	0.0176	1.483	1.116
L5P	56 L, H 62	H 62 P	4.1	0.0009	1.59	1.316
L5P	107 L, Q 113	Q 113 P	-0.6	0.0009	1.59	1.316
L5L	80 Q, K 86	Q 80 L	1	0.0001	2.102	1.618
K6E	3 K, D 10	D 10 E	3.8	0.027	2.111	1.712
K6E	87 K, D 94	D 94 E	-1.2	0.027	2.111	1.712
N6I	45 N, A 52	A 52 I	6.2	0.007	1.554	1.2
L6L	67 L, V 74	V 74 L	3.7	0.0002	2.008	1.684
L6L	52 A, L 59	A 52 L	4.3	0.0002	2.008	1.684
L6K	80 Q, K 87	Q 80 L	1	0.0017	1.884	1.47
N6T	45 N, A 52	A 52 T	-2.7	0.01419	1.491	1.261
I7I	66 I, V 74	V 74 I	2.4	0.0043	1.618	1.241
I7I	74 V, I 82	V 74 I	2.4	0.0043	1.618	1.241
L7K	52 A, K 60	A 52 L	4.3	0.0395	1.67	1.355
L7I	74 V, I 82	V 74 L	3.7	0.0018	1.704	1.346
K7E	86 K, D 94	D 94 E	-1.2	0.0045	1.971	1.573
K7K	52 A, K 60	A 52 K	-19.5	0.0003	2.059	1.632
G7N	126 G, D 134	D 134 N	6.4	3.92E-07	1.934	1.473
Y7K	52 A, K 60	A 52 Y	-7.6	0.0202	1.705	1.262
N7T	44 N, A 52	A 52 T	-2.7	0.0001	1.767	1.215
N7V	16 N, A 24	A 24 V	3.2	0.0005	1.632	1.311
N7V	44 N, A 52	A 52 V	7.8	0.0005	1.632	1.311
F7K	52 A, K 60	A 52 F	-1.5	0.0211	1.636	1.237
R7K	91 K, K 99	K 91 R	0.5	0.0111	1.446	1.2

### Applications

The database developed in this work can be used for building protein thermostability mutation libraries using different approaches like CC and also comparison of the target sequence with its homologues with higher thermostability [17, 28, 29]. In addition, it can be used for systemic analysis of modulating factors of thermostability [30–32] for different families, while thermostability modulating factors can vary from family to family [3]. Furthermore, it is noteworthy that while the thermophilic sequence belongs to microorganisms that are tolerant to harsh conditions in general and not only to temperature, these data can be used for optimization of a target sequence for new applications under other harsh conditions than temperature, like intense pH and high concentration of salts. Altogether, this database provides the most important needed data for sequence-based protein engineering and analysis for researchers to develop new analysis and engineering tools in the field of thermal stability. This database is not only useful for general industrial and research purposes but also applicable for drug design [17, 33, 34]

## Conclusions

Here we present the first release of ProtDataTherm database that contains more than 14 million protein sequences and structures belonging to microorganisms with different preferred living temperatures. All sequences and structures are labeled as psychrophilic, mesophilic and thermophilic. For ease of use, the sequences are classified based on their Pfam IDs. Users can find homologous sequences for their protein of interest by knowing its Pfam ID. This database can be applied not only for probing stability modulating factors within protein families but also for knowledge-based protein stability engineering.

### Availability

This database is available at http://profiles.bs.ipm.ir/softwares/protdatatherm. The database can be accessible free of charge for academic users on demand.
